# Temporal variation in spatial genetic structure during population outbreaks: Distinguishing among different potential drivers of spatial synchrony

**DOI:** 10.1111/eva.12852

**Published:** 2019-08-24

**Authors:** Jeremy Larroque, Simon Legault, Rob Johns, Lisa Lumley, Michel Cusson, Sébastien Renaut, Roger C. Levesque, Patrick M. A. James

**Affiliations:** ^1^ Département de Sciences Biologiques Université de Montréal Montréal Quebec Canada; ^2^ Canadian Forest Service Natural Resources Canada Fredericton New Brunswick Canada; ^3^ Royal Alberta Museum Edmonton Alberta Canada; ^4^ Laurentian Forestry Centre Natural Resources Canada Quebec City Quebec Canada; ^5^ Département de Sciences Biologiques, Institut de Recherche en Biologie Végétale Université de Montréal Montréal Quebec Canada; ^6^ Institut de biologie intégrative et des systèmes Université Laval Quebec City Quebec Canada

**Keywords:** cyclic populations, insect outbreak, SNP, spatial genetics, spruce budworm, synchrony, temporal genetics

## Abstract

Spatial synchrony is a common characteristic of spatio‐temporal population dynamics across many taxa. While it is known that both dispersal and spatially autocorrelated environmental variation (i.e., the Moran effect) can synchronize populations, the relative contributions of each, and how they interact, are generally unknown. Distinguishing these mechanisms and their effects on synchrony can help us to better understand spatial population dynamics, design conservation and management strategies, and predict climate change impacts. Population genetic data can be used to tease apart these two processes as the spatio‐temporal genetic patterns they create are expected to be different. A challenge, however, is that genetic data are often collected at a single point in time, which may introduce context‐specific bias. Spatio‐temporal sampling strategies can be used to reduce bias and to improve our characterization of the drivers of spatial synchrony. Using spatio‐temporal analyses of genotypic data, our objective was to identify the relative support for these two mechanisms to the spatial synchrony in population dynamics of the irruptive forest insect pest, the spruce budworm (*Choristoneura fumiferana*), in Quebec (Canada). AMOVA, cluster analysis, isolation by distance, and sPCA were used to characterize spatio‐temporal genomic variation using 1,370 SBW larvae sampled over four years (2012–2015) and genotyped at 3,562 SNP loci. We found evidence of overall weak spatial genetic structure that decreased from 2012 to 2015 and a genetic diversity homogenization among the sites. We also found genetic evidence of a long‐distance dispersal event over >140 km. These results indicate that dispersal is the key mechanism involved in driving population synchrony of the outbreak. Early intervention management strategies that aim to control source populations have the potential to be effective through limiting dispersal. However, the timing of such interventions relative to outbreak progression is likely to influence their probability of success.

## 1 INTRODUCTION

Spatial synchrony is the tendency of geographically separated populations to fluctuate in unison over large areas (Liebhold, Koenig, & Bjornstad, 2004) and is a common characteristic of spatio‐temporal population variability in many taxa (e.g., insects, Peltonen, Liebhold, Bjornstad, & Williams, 2002; Pollard, 1991; or mammals, Elton, 1942; Gouveia, Bjørnstad, & Tkadlec, 2016). Quantifying spatial synchrony and the mechanisms underlying it is important for improving our understanding of spatio‐temporal population dynamics (Liebhold et al., 2004), designing conservation strategies (Earn, Levin, & Rohani, 2000), informing management strategies that aim to mitigate outbreaks of pest (Régnière et al., 2001), and predicting the impacts of future climate change (Cornulier et al., 2013).

Two main contrasting theories have been proposed to explain synchrony in dynamics of spatially disjunct populations. In one theory, dispersal maintains regional synchrony owing to population flow from areas with relatively high population density to surrounding areas with low population density (Bjornstad, Ims, & Lambin, 1999; Liebhold et al., 2004). A second theory attributes spatial synchrony to spatially correlated environmental fluctuations across the landscape (i.e., the Moran effect) (Bjornstad et al., 1999; Liebhold et al., 2004; Moran, 1953). These two mechanisms are not mutually exclusive, and their relative importance has been suggested to be scale dependant (Paradis, Baillie, Sutherland, & Gregory, 2000), with dispersal being the dominant mechanism at the local scale and environmental stochasticity prevailing at larger scales (Ranta, Kaitala, & Lundberg, 1998). Despite extensive empirical and theoretical works on spatial synchrony (Liebhold et al., 2004), teasing apart the relative importance of these two processes can be difficult because they can both result in very similar patterns of spatial population dynamics (Kendall, Bjornstad, Bascompte, Keitt, & Fagan, 2000; Myers & Cory, 2013; Okland, Liebhold, Bjornstad, Erbilgin, & Krokene, 2005; Royama, MacKinnon, Kettela, Carter, & Hartling, 2005). Although dispersal is most often identified as the most likely explanation for synchrony (Franklin, Myers, & Cory, 2014; Noren & Angerbjorn, 2014; Schwartz, Mills, McKelvey, Ruggiero, & Allendorf, 2002), the Moran effect has also been implicated (Peltonen et al., 2002).

Dispersal is the more difficult of the two mechanisms to directly quantify (Ims & Andreassen, 2005). Indirect approaches using population genetic information are increasingly used to measure dispersal (Broquet & Petit, 2009). Spatial analyses of geo‐referenced molecular markers can be used to assess genetic connectivity between populations and quantify the strength, spatial scale, and effectiveness of dispersal (Baguette, Blanchet, Legrand, Stevens, & Turlure, 2013). Significant genetic differentiation between populations indicates low levels of gene flow and limited dispersal. In contrast, the absence of genetic differentiation indicates high levels of gene flow and a high degree of effective dispersal between populations.

In demographically complex outbreaking populations (i.e., populations with rapid change in population density), the strength and spatial scale of population genetic structure, as well as the inferences one can make regarding dispersal and spatial synchrony, can depend on the ecological context in which the data were collected (James et al., 2015). Here, ecological context can include standard drivers of spatial population genetic variation such as effective population size, generation time, and dispersal capacity (Charlesworth, 2009). Context can also include when during the outbreak cycle samples are collected. Samples collected during the endemic phase of an outbreak, where distant populations experience limited genetic connectivity, are likely to express relatively strong genetic spatial structure. In contrast, samples collected near the peak of an outbreak may see their original structure muted owing to the dominant signature of individuals immigrating from high‐density populations (James et al., 2015). Temporally explicit approaches to measuring spatial genetic structure are needed to better understand the spatial dynamics of outbreaking populations.

Although the two synchrony‐inducing mechanisms referred to above can be difficult to distinguish on the basis of spatial patterns alone, we expect their spatio‐temporal patterns to be unique (Box 1). Spatio‐temporal strategies that monitor changes in genetic diversity (Devillard, Santin‐Janin, Say, & Pontier, 2011) and evaluate how genetic differentiation between populations changes through time (Hoffman, Schueler, & Blouin, 2004) can be used to help us understand the underlying processes. When dispersal is driving synchrony, one expects to observe the spatial spread resulting in a genetic gradient. When the Moran effect is driving synchrony, one expects patchy genetic differentiation as a result of the independent growth of each population, with the boundaries among populations becoming less clear as the outbreak progresses (Box 1). Thus, an explicitly temporal approach allows one to overcome the limitations of the single snap‐shot approach commonly used in population genetics (Draheim, Moore, Fortin, & Scribner, 2018; Tessier & Bernatchez, 1999). However, spatio‐temporal population genetics studies of irruptive populations remain scarce in the literature (but see Berthier, Charbonnel, Galan, Chaval, & Cosson, 2006; Rikalainen, Aspi, Galarza, Koskela, & Mappes, 2012).

Box 1 The processes underlying large‐scale spatial synchrony in outbreaks of forest insect pests have fascinated population ecologists for centuries. Two central hypotheses have been proposed to describe these dynamics: (a) synchrony due to dispersal (i.e., the “epicenter hypothesis”); or (b) synchrony due to spatially correlated environmental conditions (i.e., the Moran effect). Distinguishing between these two processes can help us to better understand the spatial dynamics of population irruptions and is needed to develop effective early intervention strategies to mitigate the negative effects of pest outbreaks.

Spatio‐temporal analysis of geo‐referenced molecular markers offers a powerful tool to assess the relative weight of the two processes and to infer population dynamics over extensive geographic and temporal scales. This objective is nonetheless challenging because both the epicenter process and the Moran effect can lead to similar spatial genetic patterns when an outbreak is at or near its peak. Given the temporal contingency of outbreaks and the genetic patterns within and among populations, spatio‐temporal approaches to analyzing spatial genetic structure are required to assess the relative strength of these two processes in driving spatial synchrony. Here, we illustrate the hypothesized development of synchronous spatial genetic structure under these two processes.

The upper panel illustrates the development genetic structure under the epicenter hypothesis, and the lower panel illustrates the development genetic structure under the Moran effect, in a chronological order from the initiation of the outbreak to the peak phase (from left to right). Panel a1 (“epicenter”) shows three distinct populations, one of which is increasing in population density and spatial extent. The other two populations remain at a low density. In this case, we expect genetic structure to manifest as a gradient extending from the source population (i.e., the epicenter, panels b1 & c1) and significant isolation by distance. This rapidly spreading epicenter population can then subsume the other sites, effectively swamping out their genetic variation (d1) and leading to eventual panmixia (panel e1).

In contrast, panel a2 (“Moran effect”) shows three distinct populations that are each increasing in density and extent, presumably in response to regionally synchronous environmental conditions. Because each population's growth is independent and does not rely on dispersal from other populations, we expect the development of patchy genetic differentiation (panels b2, c2 & d2). Boundaries between populations become less clear as the outbreak progresses as a result of both the spatial expansion of the populations and dispersal among them (panel d2), leading to high levels of genetic admixture between populations (panel e2) and eventual panmixia. Genetic diversity at panmixia due to the Moran effect is (panel e2) expected to be higher than that due to the epicenter hypothesis (e1), due to the genetic contributions of multiple, versus a single, populations.

Using this framework, collection and analysis of spatial genetic data from multiple years can be used to distinguish between the processes underlying outbreak synchrony. However, successful identification of the dominant process requires that sampling occurs early enough during the outbreak, before panmixia is reached. After panmixia is reached (i.e., panels e1 and e2), the genetic legacy of the previous outbreak collapse may no longer be detectable. The specific rate at which these historical legacies in spatial genetic structure fade is likely a function of multiple demographic parameters such as effective population size, dispersal capacity, and genetic diversity within the population of interest. The precise roles of these factors remain to be further investigated.

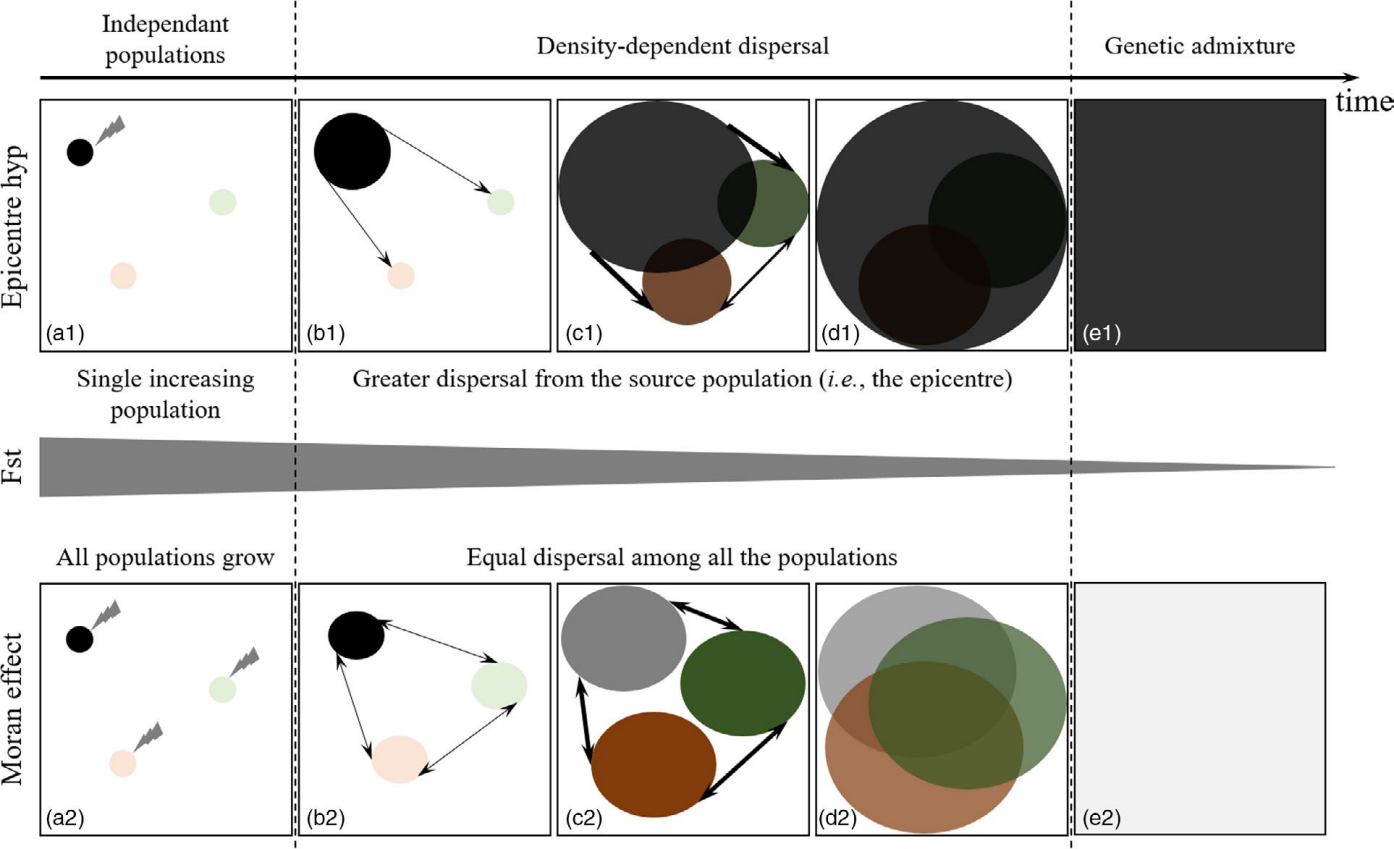

The spruce budworm (SBW; *Choristoneura fumiferana* Clemens) provides an excellent example of spatially synchronous population dynamics. The SBW is a univoltine native lepidopteran that periodically outbreaks (every ~35 years) and defoliates large areas (>10^6^ ha) of balsam fir (*Abies balsamea* (L.) Mill) and spruce (*Picea* spp.) forests in North America (Royama, 1984). SBW populations exhibit both local epicentric growth (Greenbank, Schaefer, & Rainey, 1980; Hardy, Lafond, & Hamel, 1983) and regional‐scale synchronization (up to 500,000 km^2^) during outbreaks (Royama, 1984). The resulting economic consequences for forest industries and forestry‐dependent communities are severe (Chang, Lantz, Hennigar, & MacLean, 2012).

Large‐scale spatial synchrony of SBW outbreaks is a common feature in North American forests, particularly in the eastern part of its range (Blais, 1983; Pureswaran, Johns, Heard, & Quiring, 2016; Williams & Liebhold, 2000). However, despite a century of research, there remains opportunity to improve our understanding of these large‐scale population processes, including how dispersal contributes to spatial synchrony (Anderson & Sturtevant, 2011; James et al., 2015; Myers & Cory, 2013; Pureswaran et al., 2016).

In this paper, we use multi‐year spatial genetic data, covering the extent of an ongoing SBW outbreak, to investigate the spatial processes involved in synchronous outbreak dynamics. Specifically, we investigate whether outbreaking populations are synchronized as a result of (a) exposure to a common regional disturbance (i.e., the Moran effect, Moran, 1953; Royama et al., 2005) resulting in demographically independent populations and thus, high levels of population genetic differentiation (Box 1); or (b) dispersal of adults moths (i.e., the "epicenter hypothesis," Hardy et al., 1983; Peltonen et al., 2002; Williams & Liebhold, 2000) leading to low genetic differentiation among populations (Box 1). We also assess how the relative support for these two hypotheses evolved over a 4‐year period. Distinguishing between the epicenter and Moran hypotheses is critical for large‐scale forest management strategies against the SBW in Canada and the United States (Pureswaran et al., 2016).

## 2 MATERIALS AND METHODS

### 2.1 Study area

**Figure 1:**
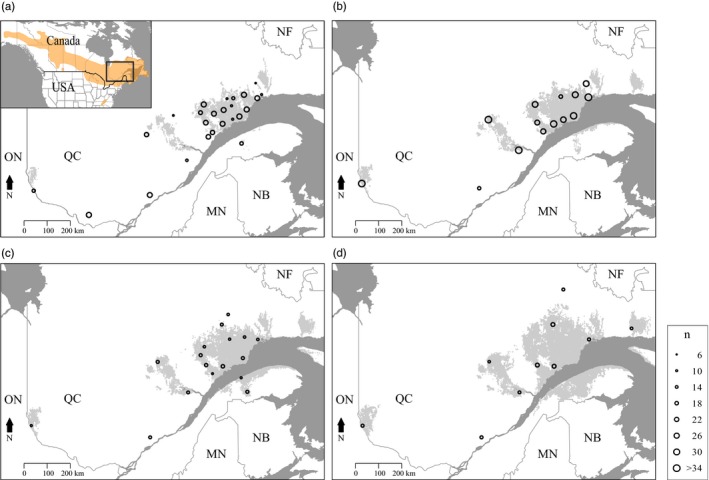
Spruce budworm distribution range, study area, and sampling sites. Sites sampled in (a) 2012; (b) 2013; (c) 2014; and (d) 2015 are represented by points whose size is proportional to the site's sample size (*n*). Spruce budworm distribution range (adapted from Picq et al., 2018) is displayed in orange, and defoliation areas observed the year of sampling are represented in gray

The study was conducted in the boreal and mixed‐boreal forest (Rowe, 1972) in Quebec, Canada (398,000 km^2^, Figure 1). In 2006, a new outbreak was detected on the north shore of the Saint Lawrence River in Quebec (Bouchard & Auger, 2014). Since then, the area affected has increased to >8.2 million ha in Quebec (Ministère des Forêts, de la Faune et des Parcs [MFFP], 2018) and is currently moving toward other jurisdictions (e.g., New Brunswick and Maine, where larvae densities are still low) to the south. Spruce budworm larvae were collected over a 2‐week period in June, from 25 locations in 2012 (*n* = 527), 14 locations in 2013 (*n* = 420), 17 locations in 2014 (*n* = 260), and 10 locations in 2015 (*n* = 163). In 2012, sites were selected to cover all the outbreak patches. Not all sites were identical from one year to the next. Some sites were discarded due to low larvae density or trouble accessing the site, but close (i.e., <10 km) replacement sites were selected when possible. When substitute sites were selected, we considered the original and the substitute site to be a single site through time (Table S1).

Each year, we sampled only larvae (no moths) from all defoliated zones to ensure that all individuals were associated with their site and the possibility of migrants from outside the study area would not affect the results. Many sites were sampled successively in all years, whereas some were sampled for only a single year (Figure 1). Larvae were collected on live foliage from branch tips (Dobesberger & Lim, 1983) on both spruce and fir when they were both present at the site. Multiple branches from multiple trees were sampled from each site to reduce the probability of sampling related individuals from the same clutch. Larvae were transported on branches in paper bags and were reared in the laboratory on synthetic diet (McMorran, 1965) until moth eclosion. Emerging moths were placed into 1.5‐mL tubes and stored at −80°C until DNA extraction. Genomic DNA was extracted from 6 to 33 individuals per site (total of 1,370 individuals) using the Qiagen DNA Blood and Tissue Kit.

### 2.2 Sequencing

All DNA samples were prepared for genotyping‐by‐sequencing (GBS, Elshire et al., 2011) using the methods described in Brunet et al. (2017). *PstI‐MspI* GBS libraries (96 plex) were prepared by the Institut de Biologie Intégrative et des Systèmes (IBIS) at Université Laval (Quebec City, QC) using the protocol of Poland, Brown, Sorrells, and Jannink (2012). Following final amplification of the pooled, adapter‐ligated restriction fragments, 600 ng of each amplified library was normalized to remove the repetitive fraction by treatment with duplex‐specific nuclease (Zhulidov et al., 2004). Finally, an additional PCR step using a selective reverse primer extending a single base (C) into the insert past the 3′ restriction site was used to selectively amplify one‐quarter of the total number of fragments, thereby increasing the read depth of sequenced fragments (Sonah et al., 2013). Single‐end sequencing (100 bp reads) of these libraries was then performed with an Illumina HiSeq2000 (McGill University‐Génome Québec Innovation Centre, Montreal, QC).

Bioinformatic processing of reads was performed using the Fast‐GBS pipeline (Torkamaneh, Laroche, Bastien, Abed, & Belzile, 2017). The Fast‐GBS pipeline includes demultiplexing, trimming, mapping, and variant calling steps to process genotyping‐by‐sequencing samples and provide highly accurate genotyping. This pipeline has been shown to yield the highest accuracy compared to the other pipelines (Torkamaneh, Laroche, & Belzile, 2016). After demultiplexing and adapter trimming, reads less than 50 bp were discarded. A total of 4.162 gigabases of reads were aligned on the spruce budworm reference genome (bw6 version, Dupuis et al., 2017; Picq et al., 2018). Alignment of 3.141 gigabases of reads was performed using the Burrows–Wheeler Aligner (BWA, Li & Durbin, 2010) with the *mem* algorithm and yielded a 75% mapping success. SNP calls were made as part of the Fast‐GBS pipeline using Platypus (v0.8.1, Rimmer et al., 2014). Minimum read depth was set to eight reads. Only bi‐allelic SNPs, with a maximum of 50% missing genotypes throughout all samples, were retained. Individuals with more than 50% missing genotypes were removed. Finally, as some methods cannot handle missing data, missing genotypes were imputed using the software BEAGLE (v3.3.2, Browning & Browning, 2016) which replaces missing genotypes with the most frequently observed genotype associated with proximal SNP loci. Variants with *R* ^2^ (i.e., imputation accuracy) <.4 were removed as advised by Browning and Browning (2016).

### 2.3 Filtering SNP loci

SNPs with a minor allele frequency (MAF) <5% were removed to exclude putative sequencing errors and keep only the most informative SNPs (Marees et al., 2018). Similarly, SNPs in high linkage disequilibrium (LD) at a threshold of *r* ^2^ ≥ .2 were discarded to remove highly correlated variants that add minimal extra information and could overly influence some methods (Price et al., 2008; Zou, Lee, Knowles, & Wright, 2010), using the *snpgdsLDpruning* function of the *SNPRelate* package (Zheng et al., 2012) in R (R Core Team, 2017). SNPs showing deviation from expected Hardy–Weinberg equilibrium (HWE) in more than 15% of the sites were also removed as deviation from HWE can be indicative of genotyping errors (Anderson, Epperson, et al., 2010; Anderson, Pettersson, et al., 2010), null alleles (Brookfield, 1996), or selection (Wittke‐Thompson, Pluzhnikov, & Cox, 2005). HWE was calculated using the *hw.tes*t function of the *pegas* package in R (Paradis, 2010) and using a Bonferroni correction for multiple comparisons. Finally, as our aim was to quantify the neutral evolutionary process of gene flow, we removed all SNPs identified as potentially under selection (Beaumont & Nichols, 1996) using the *pcadapt* function of the *pcadapt* package in R (Luu, Bazin, & Blum, 2017).

### 2.4 Genetic diversity

For each site and for each year, we calculated observed (*Ho*) and expected (*He*, heterozygosity expected under Hardy–Weinberg equilibrium that accounts for both the number and the evenness of alleles) heterozygosity, rarefied allelic richness (*Ar*, El Mousadik & Petit, 1996), inbreeding coefficient (*Fis*), and total number of alleles (*n.all*) using the *hierfstat* R package (Goudet & Jombart, 2015). The degree of structuring between subpopulations (*Fst*) was computed within each year also using *hierfstat*.

#### 2.4.1 Inter‐individual genetic distances

We calculated inter‐individual genetic distances using principal components analysis (PCA). We first created multiple principal components (PC) using a matrix of allele occurrence (0, 1, or 2) using *adegenet* (Jombart, 2008) and then derived a distance matrix from the Euclidean distances between individuals in the multidimensional space created by the first 64 PC axes (Shirk, Landguth, & Cushman, 2017).

### 2.5 Spatio‐temporal genetic variation

To examine the effects of space (sampling sites) and time (year of sampling) on patterns of genetic variation, we conducted a permutation‐based multivariate analysis of variance using the function *adonis* of the *vegan* package (Oksanen et al., 2017) in R. This method partitions sum of squares for distance matrices in a manner similar to AMOVA, but allows for both nested and crossed factors. We tested for the effects of space (sampling sites) and time (year of sampling) as crossed factors on the matrix of individual genetic distances. Statistical significance was assessed using 9,999 permutations. Given the signal of temporal variability observed (see Results), subsequent analyses were done for each year separately.

As the number of sites and the number of individuals sampled per site varied among years, we performed a rarefied bootstrap to standardize for the minimum number of sites per year and the minimum number of individuals per site to ensure that there was no bias due to the unbalanced sampling. We subsampled the data keeping only 10 sites per year and 8 individuals per site and performed the AMOVA. We repeated this procedure 9,999 times.

### 2.6 Clustering

If populations are not connected, their growth will be independent as it does not rely on dispersal from other populations. In this case, we expect individuals to cluster into genetic groups that correspond to geographic provenance. We searched for genetic groups using discriminant analysis of principal components (DAPC) implemented in the *adegenet* (Jombart, 2008) package in R. DAPC maximizes differences among clusters while minimizing variation within. DAPC does not rely on a particular population genetic model, such as Hardy–Weinberg equilibrium, which is unrealistic for outbreaking populations (Whitlock, 1992). For each year, we applied the function *find.clusters* to determine the number of potential clusters. Minimization of a Bayesian information criterion (*BIC*) was used to identify the most probable number of clusters (*K*) present in the data. DAPC provides membership probabilities to these clusters for each individual, which we examined for geographic structure.

### 2.7 Isolation by distance

If populations are connected through dispersal, then the genetic similarity between two populations is a function of the geographical distance between them. Consequently, genetic and geographic distances are expected to be correlated (i.e., isolation by distance, IBD; Wright, 1943). We tested for IBD by testing the correlation between genetic distance and the geographic Euclidean distance between all pairs of individuals. Significance of the correlation between the two distance matrices was assessed by carrying out a Mantel test using the *mantel.randtest* function of the *ade4* (Dray & Dufour, 2007) R package with 9,999 permutations.

### 2.8 Cryptic spatial structure

When populations exchange individuals, they tend to become genetically similar. The integration of geographic and genetic information can improve our ability to identify weakly differentiated populations and can provide accurate spatial locations of cryptic clusters or genetic barriers (Storfer et al., 2007). Given the weak overall structure (i.e., clusters and IBD; see below), we also tested for cryptic spatial genetic structure within each year using spatial principal component analysis (sPCA; Jombart, Devillard, Dufour, & Pontier, 2008). This spatially explicit multivariate method employs Moran's index (*I*) of spatial autocorrelation (Moran, 1948) to detect global structures (Jombart et al., 2008). We used the *spca* function implemented in the *adegenet* (Jombart et al., 2008) R package. We used the inverse distance method for weighting connectivity in the network, given that: (a) Sampling sites were unevenly spread over the study area; (b) we had no a priori hypothesis about their connectivity. Significance was assessed using permutation test (*n* = 9,999) (Jombart et al., 2008). The individuals’ scores of the first principal component were geographically mapped and interpolated to reveal spatial patterns of interest.

## 3 RESULTS

### 3.1 Genotyping

Sequence processing through the fast‐GBS pipeline (Torkamaneh et al., 2017) resulted in 73,960 high‐quality SNPs (mean read depth ± *SD* = 3,018 ± 19,214 – range: 8–1,352,493, mean fraction of missing genotype per SNP ± *SD* = 0.68 ± 0.32). These SNPs were identified using 1,228 individual larvae (*n*_2012_ = 464, *n*_2013_ = 386, *n*_2014_ = 223, *n*_2015_ = 155) that satisfied the missing genotype criterion (<50%; mean fraction of missing genotype per individual ± *SD* = 0.22 ± 0.19). Most SNPs (73,489; 99.4%) had at least one missing genotype which was then imputed (mean fraction of missing genotype per SNP ± *SD* = 0.17 ± 0.15; range = 0–0.49; Figure S1) and 0.7% (480) of imputed SNPs were removed when filtering for *R* ^2^. Many SNPs (69,045; 94%) were discarded when filtering for MAF and LD. Of the remaining SNPs, 4.1% (182) deviated from HWE in more than 15% of the sampling sites. About 16% (691) of the remaining SNPs were detected as potentially under selection and removed. In total 3,562 SNPs met our strict selection criteria and were retained for final analysis.

### 3.2 Genetic diversity

Over the four years of sampling, average observed heterozygosity (*Ho*) was 0.172 (±0.013) and average expected heterozygosity (*He*) was 0.211 (±0.011), with a mean allelic richness per locus (*Ar*) of 1.738 (±0.033). The total number of alleles (*n.all*) was 6,774 (±274), and the inbreeding coefficient (*Fis*) was 0.166 (±0.028). *He*, *Ho*, *Ar*, *n.all,* and *Fis* varied through time (Table 1, Figure S2). Global *Fst* remained low for all years examined (0.002–0.0046; Table 1). Detailed information per year and per sampling site can be found in the Supporting Information (Table S1).

**Table 1:** Annual summary of sampling sites. Annual (±*SD*) sample size (*n*), observed heterozygosity (*Ho*), expected heterozygosity (*He*), allelic richness (*Ar*), total number of alleles (*n.all*), and inbreeding coefficient (*Fis*) averaged over all sites of a given year*.* Global *Fst* and mean inter‐site Euclidean distance (*D*_intersite_ in km) computed for each year are also shown

Year	*n*	*Ho*	*He*	*Ar*	*n.all*	*Fis*	*Fst*	*D*_intersite_
2012	464	0.168 ± 0.013	0.207 ± 0.011	1.721 ± 0.040	6,705 ± 352	0.168 ± 0.022	0.0046	273 ± 241
2013	386	0.184 ± 0.016	0.217 ± 0.007	1.762 ± 0.016	7,039 ± 58	0.148 ± 0.045	0.0020	299 ± 250
2014	223	0.169 ± 0.007	0.211 ± 0.009	1.736 ± 0.026	6,645 ± 159	0.170 ± 0.012	0.0023	285 ± 230
2015	155	0.171 ± 0.006	0.216 ± 0.007	1.752 ± 0.015	6,794 ± 107	0.180 ± 0.016	0.0022	424 ± 262

**Figure 2:**
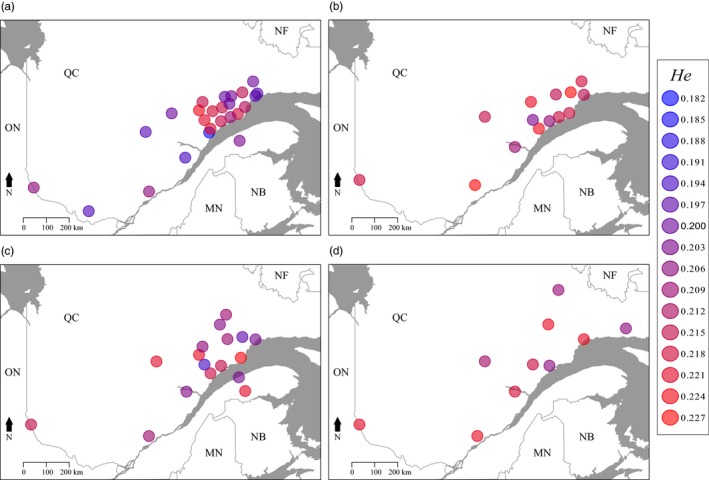
Expected heterozygosity (*He*) for each sampling site in (a) 2012; (b) 2013; (c) 2014; and (d) 2015. Heterozygosity generally increased as the outbreak progressed (Table 1). The color gradient is proportional to the *He* level with the lowest values in blue and the highest in red

We found that *He* varied spatially and temporally (Figure 2). In 2012, western sites showed low *He* values while eastern sites showed a mix between high and low *He* values (Figure 2a). From 2013 to 2015, *He* was higher and more homogeneously distributed (Figure 2a–d).

### 3.3 Spatio‐temporal genetic variation partitioning

Using a permutation‐based multivariate analysis of variance, we found a significant interaction effect among years and sites (*F*_25, 1,162_ = 1.32, *p* < 10^–4^; Table 2) on spruce budworm genetic variation. The rarefied bootstrap approach showed that with a standardized number of sites per year and number of individuals per site, the effect of the year and the site were significant in 100% of the 9,999 replications. The effect of the interaction was significant in 70% of the replications. This indicates that the processes generating spatial genetic structure were dynamic, rather than static, over the four years we analyzed. Consequently, analyses were undertaken for each year separately (Figures 2, 4, and 5).

**Table 2:** Permutation‐based multivariate analysis of variance to determine the percentage of variance explained by the effects of sampling sites, year of sampling and their interaction on the matrix of PCA‐based genetic distance. Significance was assessed using 9,999 permutations

	*df*	SS	MS	*F* value	*R* ^2^	*p*
Year	3	1518	505.89	15.11	.034	<10^–4^
Site	37	2,149	58.09	1.73	.049	<10^–4^
Year × site	25	1,106	44.25	1.32	.025	<10^–4^
Residuals	1,162	38,917	33.49			
Total	1,227	43,691				

Abbreviations: MS, mean square; SS, sum of squares.

### 3.4 Clustering

**Figure 3:**
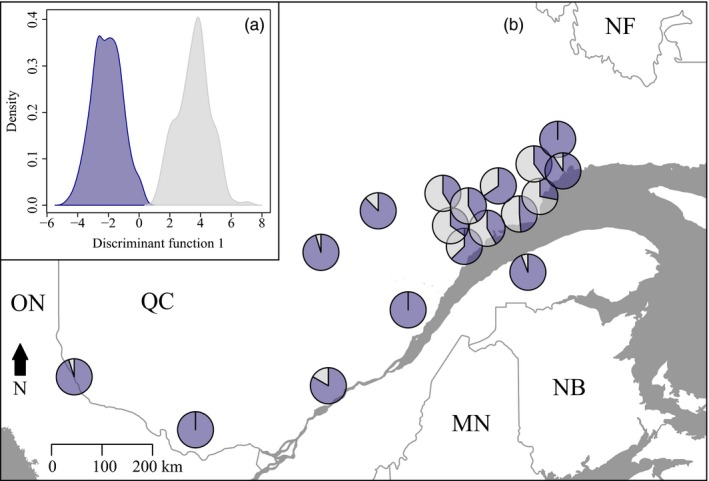
(a) Assignment of 2012 (the only year with a clear signal for *K* = 2) individuals by the discriminant analysis of principal components (DAPC) to the two (blue and gray) genetic clusters (pairwise *Fst* = 0.007) and (b) map of sampling sites illustrating membership to the two identified genetic clusters. Sampling sites less than 40 km apart were merged to increase visibility (original figure can be found in Figure S7)

Using DAPC, a *BIC* minimum was observed for *K* = 2 for 2012 supporting two genetic clusters (Figure S3). Genetic structure between these two clusters was weak (*Fst* = 0.007) but significant (*p* < 10^–3^). The two clusters initially identified in 2012 were no longer present from 2013 to 2015; instead, *K* = 1 had the greatest support (Figure S3). We found some evidence for geographic structure within the two clusters in 2012 (Figure 3b). Southwestern sites were mostly composed of individuals belonging to the same genetic cluster, whereas northeastern sites were composed of a mixture of individuals assigned to both clusters. Using a subsample of 150 individuals that had been sexed, we verified that the clustering was not based on sex (not shown).

### 3.5 Isolation by distance

**Figure 4:**
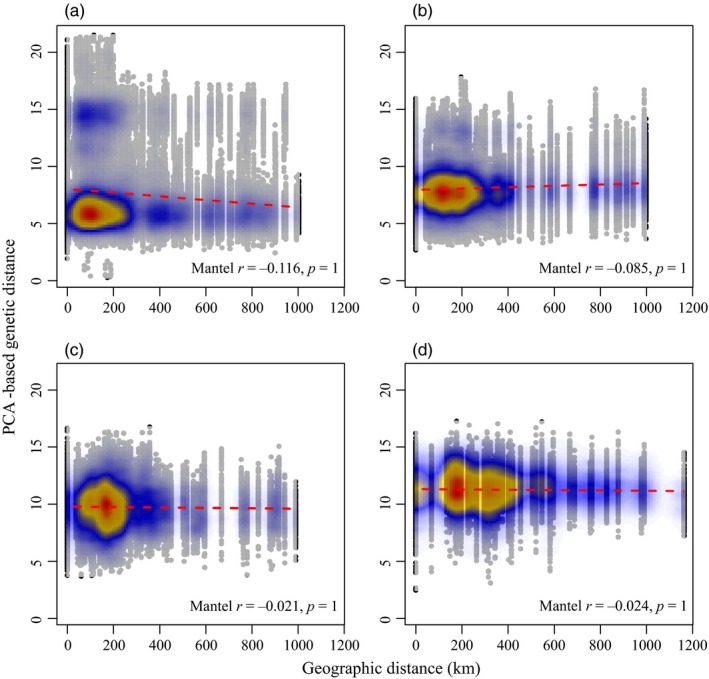
Isolation by distance (IBD) plots for each year. Scatterplots show the relationship between inter‐individual genetic distances (PCA‐based genetic distance, Shirk et al., 2017) and geographic distances to test for the presence of IBD in (a) 2012; (b) 2013; (c) 2014; and (d) 2015. Colors represent the relative density of points, with warmer colors indicating higher densities, while the dashed line shows the linear regression between the two distance matrices. The Mantel coefficient of correlation between geographic and genetic distances, as well as the associated *p*‐value, is shown for each year

For all years examined, we found no evidence for significant individual‐level IBD (all *p* = 1, Figure 4). The point density for 2012 showed two clusters of individuals with the same level of genetic differentiation, suggesting the presence of two populations that seemed to be genetically distinct (Figure 4a), which supports our clustering result described above. These clusters were no longer present from 2013 to 2015 (Figure 4b–d) and the variance of the genetic distance decreased, suggesting a genetic homogenization as the outbreak progressed.

### 3.6 Cryptic spatial structure

**Figure 5:**
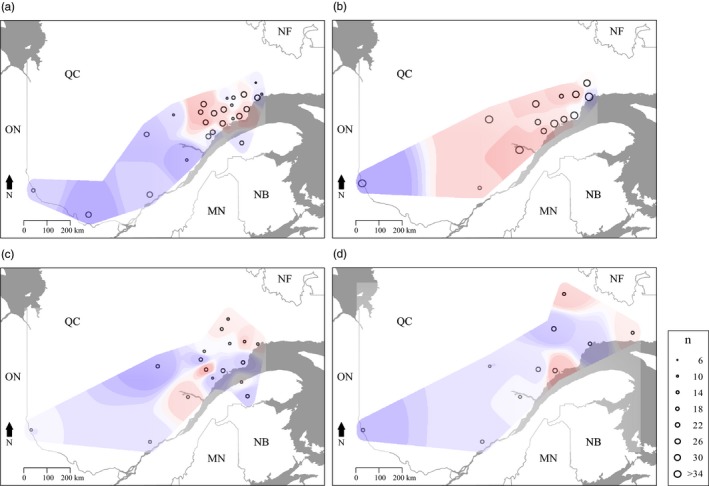
Interpolated spatial genetic structure through time, based on sPCA. Spatial interpolation of individual scores according to the first positive eigenvalue of the sPCA in (a) 2012; (b) 2013; (c) 2014; and (d) 2015. Significance was assessed using 9,999 permutations. The color gradient indicates the degree of difference between individuals. Maximum differentiation is between dark blue and red. The extent of the interpolated region was defined as the concave hull polygon encompassing all the sampling sites for a given year with an external buffer of 5 km. Sampling sites are represented by points whose size is proportional to the site's sample size (*n*)

We identified significant global cryptic spatial structure for each year (all *p* ≤ .01). However, the first positive eigenvalue, which represents global spatial genetic structure, decreased in magnitude from 2012 to 2015 compared to the other eigenvalues (Figure S4), indicating a genetic homogenization over the years. The scores of the first positive eigenvalue have been interpolated and displayed on the area map (Figure 5).

In 2012, our results regarding global structure indicated that individuals belonged to two genetically homogeneous clusters, one large cluster distributed all over the study area, and a second one only present at the northeast (Figure 5a). Individuals on the south shore of the Saint Lawrence River belonged to the more extensive genetic cluster and were different from the nearest individuals of the north shore. In 2013 (Figure 5b), we found clinal variation from west to east, with the notable exception of a group of individuals at the east that were similar to some western individuals. In 2014, the first positive eigenvalue revealed a complex pattern. Two genetic clusters were found with no clear geographical delimitation (Figure 5c). One cluster was spread all over the study area while the smaller one showed a patchy distribution, with most of its individuals located in the center and east of the study area. Individuals on the south shore of the Saint Lawrence River were assigned to both clusters. The pattern found in 2015 was similar to that of 2014 (Figure 5d).

## 4 DISCUSSION

Spatial synchrony is a fundamental but poorly understood, component of spatial population dynamics. Regionally, synchronous population fluctuations can be caused by dispersal or spatially correlated environmental conditions (Moran, 1953; Peltonen et al., 2002; Ranta, Kaitala, Lindstrom, & Helle, 1997; Ranta, Kaitala, Lindstrom, & Linden, 1995; Royama, 1984), although in general, we do not know the relative importance of each of these factors and how they vary through space and time (Liebhold et al., 2004). Distinguishing these mechanisms and their effects on synchrony can help us to better understand spatial population dynamics, design conservation, and management strategies, and predict climate change impacts.

In 2006, a new SBW outbreak was detected on the north shore of the Saint Lawrence River that has since spread to over 8 million hectares in size. Using 4 years of genetic data covering the majority of the outbreak area (i.e., 398,000 km^2^), we quantified temporal changes in spruce budworm spatial genetic structure and estimated gene flow through space and time. Our goal was to use spatial genetic information to better understand the relative importance of dispersal to spatial outbreak synchrony (Box 1) and to contribute to improved budworm management. We found evidence for fast genetic admixture, long‐distance dispersal, and increases in genetic diversity. Together, these findings suggest a more important role of dispersal relative to autocorrelated environmental variation in the spatial synchrony of this species during an outbreak.

### 4.1 Spatio‐temporal connectivity in SBW

Disentangling the effects of epicenter and Moran processes on spatially synchronous SBW population dynamics is challenging because both processes can lead to similar genetic patterns when the outbreak is at or near its peak (Box 1). We found greater support for the epicenter hypothesis for the period covered by this study. In 2012, a single genetic cluster was found over the entire study area whereas a second one was restricted to a smaller region in the northeast, overlapping with the first cluster (Figures 3, 4, 5). We interpret these results to indicate that an isolated population, possibly the legacy of the previous outbreak collapse (James et al., 2015) (i.e., the larger cluster), expanded to encompass a second population (i.e., the second, smaller cluster). This process of an initial isolated population expanding via dispersal fits the pattern expected from an epicentric population dynamic (Box 1).

We cannot entirely reject the possibility that the first cluster may have been present before the outbreak and the small cluster represents immigrants from outside the study area. However, we collected samples from all defoliation zones (Figure 1) to minimize the possibility that migrants from outside the study area could affect our results. Additionally, temporal patterns in defoliation surveys (Figures S6 and S8, Ministère des Forêts, de la Faune et des Parcs [MFFP] 2018), and the spatial extent of historical defoliation support the first of our proposed explanations (Brown, 1970). That is, defoliation was first observed in the western part of Quebec, further indicating that dispersal from this area to the east may account for the observed topological complexity in spatial genetic structure. If the Moran effect was the dominant processes in the outbreak spread, eastern and western populations would have grown simultaneously (Box 1) and one would expect eastern individuals to form a distinct group. Instead, eastern individuals are found together with western individuals in the east. This suggests that individuals dispersed from the western part of the province at the beginning of the outbreak, that is, the outbreak epicenter, and settled in the east, providing support for the epicenter hypothesis. Such directional dispersal could have significant effects on the development and persistence of spatial genetic structure of highly mobile species such as the SBW, although little is known about how to accurately capture spatial genetic patterns generated in this way.

The decline in the strength of spatial genetic structure from 2012 to 2015 (Figure 5, Figures S3 and S4) also supports the importance of dispersal to outbreak synchrony. The important role of dispersal is also illustrated by rapid homogenization of genetic diversity (e.g., *He*). Although *He* was spatially clustered in 2012, from 2013 to 2015 *He* became increasingly homogeneously distributed.

### 4.2 Long‐distance dispersal

Rapid change in SBW genetic composition on the south shore between 2012 and 2014 indicates long‐distance dispersal and successful establishment; that is, the eggs laid by dispersing females successfully hatched (Figure 5). Previous studies have shown that SBW moths can disperse up to 20 km with a maximum recorded passive dispersal distance of 450 km (Greenbank et al., 1980). More recently, Boulanger et al. (2017) documented a mass dispersal over more than 200 km in 2013 from the north to the south shore of the Saint Lawrence River using weather surveillance radar data. Although our ability to infer source populations and dispersal capacity is somewhat constrained by the limited genetic structure we observed (Muirhead et al., 2008), we have demonstrated for the first time that long‐distance dispersal events result in successful establishment, and hence can be considered as effective dispersal.

Long‐distance dispersal is common in Lepidoptera (Showers, 1997) and has a disproportionately high influence on gene flow and genetic structure (Clobert, Baguette, Benton, Bullock, & Ducatez, 2012; Jordano, 2017). It can connect disparate populations, allowing for genetic connectivity and range expansion (Baguette & Schtickzelle, 2006; Ronce, 2007; Trakhtenbrot, Nathan, Perry, & Richardson, 2005). Comparative studies suggest that species that can disperse farther tend to be synchronized over larger areas (Paradis, Baillie, Sutherland, & Gregory, 1999; Sutcliffe, Thomas, & Moss, 1996). In SBW, several long‐distance mass exodus flights of millions of individuals have been observed (Boulanger et al., 2017; Dickison, 1990; Greenbank et al., 1980; Sturtevant et al., 2013). Such events have the potential to initiate new epicenters (Clark, 1979) and to synchronize the dynamics of different SBW populations over large geographic areas (Muenkemueller & Johst, 2008; Peltonen et al., 2002). In their study, Bouchard, Régnière, and Therrien (2017) suggested that dispersal may play a prevalent role in SBW outbreak synchrony once outbreak levels have been reached. Through the growing phase of the outbreak, long‐distance dispersal events may have contributed to the observed genetic admixture and may still be contributing to the spread of the outbreak over a large territory in Quebec and adjacent provinces and U.S. states (e.g., New Brunswick and Maine).

### 4.3 The challenge of cyclic populations

The application of population genetic approaches to the study of cyclic species is challenging because of frequent deviations from theoretical predictions due to highly variable population dynamics and dependence on ecological context (James et al., 2015). The observed decline in spatial genetic structure through time with outbreak spread fits well with the predictions of James et al. (2015) that spatial genetic structure decreases as the outbreak spreads and connectivity increases. These results indicate that temporal approaches are needed to characterize the role of population periodicity on the spatial patterns in neutral genetic variation (Box 1).

Spatial patterns of genetic diversity are the result of multiple factors that interact through space and time. Molecular studies usually present a single snap‐shot description of spatial genetic structure, assuming temporal stability (Draheim et al., 2018; Tessier & Bernatchez, 1999). It is increasingly recognized that temporal aspects matter in population genetics but also in landscape genetics studies (Anderson, Epperson, et al., 2010; Anderson, Pettersson, et al., 2010; Martensen, Saura, & Fortin, 2017; Skoglund, Sjodin, Skoglund, Lascoux, & Jakobsson, 2014). However, the utility of temporal approaches in molecular studies of wild populations has mostly been limited to the comparison of historical and contemporary samples (Ruggeri et al., 2012). We have demonstrated here that temporal sampling can be used to identify the relative importance of dispersal and Moran effect in the regulation of outbreak synchrony. However, the spatial genetic structure of noncyclic populations can also quickly evolve in connection with the functional connectivity as landscape and environmental conditions change. For instance, Watts et al. (2015) showed that a decrease in snowpack is associated with reduced colonization and less gene flow in boreal chorus frogs (*Pseudacris maculata*). Time‐structured data thus offer a new dimension of information that enables identification and better understanding of demographic changes. Such temporal analysis can also highlight the risks of failing to consider temporal variability when inferring population demographic parameters (e.g., dispersal capacity) on the basis of a static assessment of spatial genetic structure. Temporal investigations of the spatial genetic structure of cyclic and noncyclic species could be instructive in improving our understanding of how their dynamics are influenced by changes in functional connectivity, that is, by changes in their habitat conditions, an increasingly important issue given the fast environmental changes induced by global warming and the loss and fragmentation of habitat (Fahrig, 2003).

### 4.4 Conclusion

Through novel spatio‐temporal analysis of genetic data, we found support for the epicenter hypothesis as the central driver of synchronous SBW dynamics in Quebec. We also found evidence for long‐distance dispersal (>140 km), which indicates that SBW moths can not only travel large distances (e.g., Greenbank et al., 1980), but can also successfully reproduce at landing sites. Knowledge of the importance of effective dispersal to outbreak synchrony is essential to the development and implementation of “early intervention” forest management strategies (Régnière et al., 2001) that aim to mitigate forest losses due to defoliation.

In Quebec, SBW populations are currently well connected and exhibit genetic panmixia. Such panmixia poses a challenge to forest managers seeking to contain the spread of SBW outbreaks from Quebec into other areas, such as Atlantic Canada. Early intervention strategies (Pureswaran et al., 2016) entail suppressing global population growth by finding and treating emerging local “hot spots.” One of the key factors contributing to hot spots in currently unaffected areas is moths’ dispersal from Quebec. Using genetic data, our study further confirms that SBW moths can disperse very long distances (e.g., Boulanger et al., 2017; Greenbank et al., 1980). In addition, we also demonstrate that this dispersal contributes to local population growth; that is, dispersal is “effective.” However, it remains to be seen whether the demographic impact of dispersers can overwhelm control efforts in areas, especially in relatively low density areas where populations have yet to establish. Characterization of dispersal and, in particular, the frequency distribution of the dispersal distances, could help to better delineate the range and distribution of dispersing moths throughout a region, which could help to guide survey efforts for both population studies and control efforts.

Our ability to distinguish between different drivers of outbreak synchrony will vary depending on the characteristics of the species being studied. For example, our approach may be sensitive to species‐specific factors such as effective population size, generation time, dispersal capacity, outbreak frequency, and landscape heterogeneity, as all of these factors influence the spatial distribution of genetic variation (Gauffre et al., 2015; Landguth et al., 2010; Row, Wilson, & Murray, 2016). Further exploration of the temporal dynamics of spatial genetic structure during population outbreaks under different contexts remains a promising avenue for future research. Simulation‐based approaches using spatially explicit demo‐genetic models (e.g., Nemo, Guillaume & Rougemont, 2006; or CDMetaPOP, Landguth, Bearlin, Day, & Dunham, 2017) hold great promise to isolate and quantify the relative effects of these different factors on the development of spatial genetic structure and spatial synchrony, and to refine our understanding of what drives these large‐scale spatial ecological phenomena.

## CONFLICT OF INTEREST

None declared.

## Data Availability

One Genalex file representing the filtered SNPs data set used for analyses and the reference genome version used in this manuscript are available at the Dryad Digital Repository: https://doi.org/10.5061/dryad.1vr6g3f (embargoed until one year after the article is published). During the embargo period, the reference genome can be made available upon request to M. Cusson (michel.cusson@canada.ca). Larroque et al., 2019.
