# Supplementary material for: Temporal variation in spatial genetic structure during population outbreaks: Distinguishing among different potential drivers of spatial synchrony
Source: Evol Appl. 2019 Aug 24;12(10):1931–45. doi: 10.1111/eva.12852 (PMC6824080; doi:10.1111/eva.12852)
Supplement: Supplementary file 1 [file EVA-12-1931-s001.docx]

**Supporting information for**

**Temporal variation in spatial genetic structure during population outbreaks: distinguishing among different potential drivers of spatial synchrony**

**Figure S1** Distribution of the missing values per selected SNP satisfying the read depth and missing genotypes criterion. The mean fraction of missing genotype per SNP ± SD was 0.17 ± 0.15 – range: 0-0.49.

**
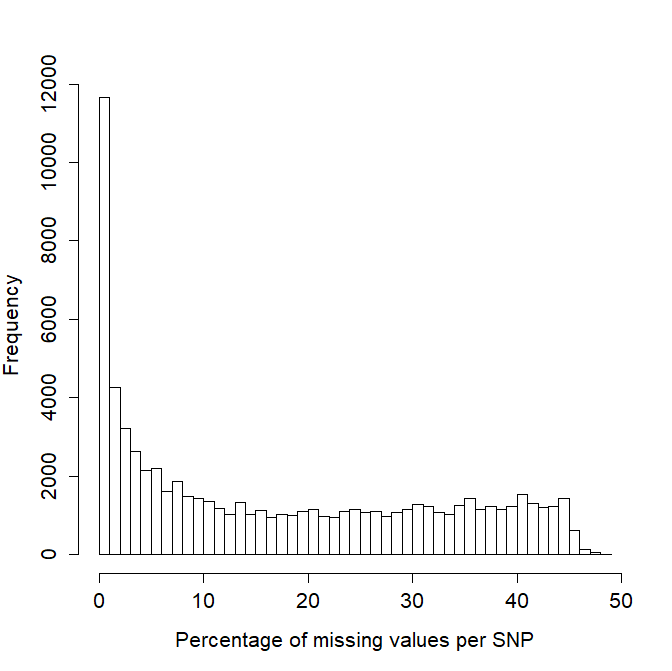
**


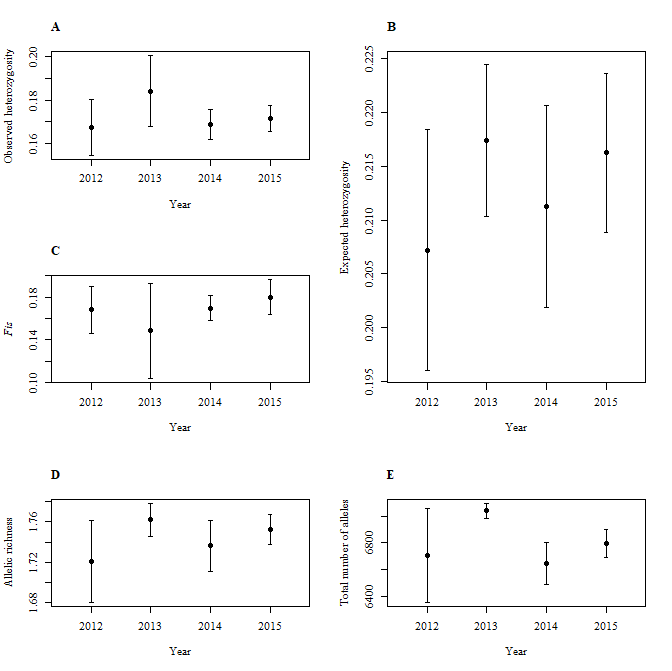


**Figure S2** Temporal evolution of the genetic diversity indices from 2012 to 2015. Annual (± SD) A) observed heterozygosity, B) expected heterozygosity, C) *Fis*, D) allelic richness and E) total number of alleles.


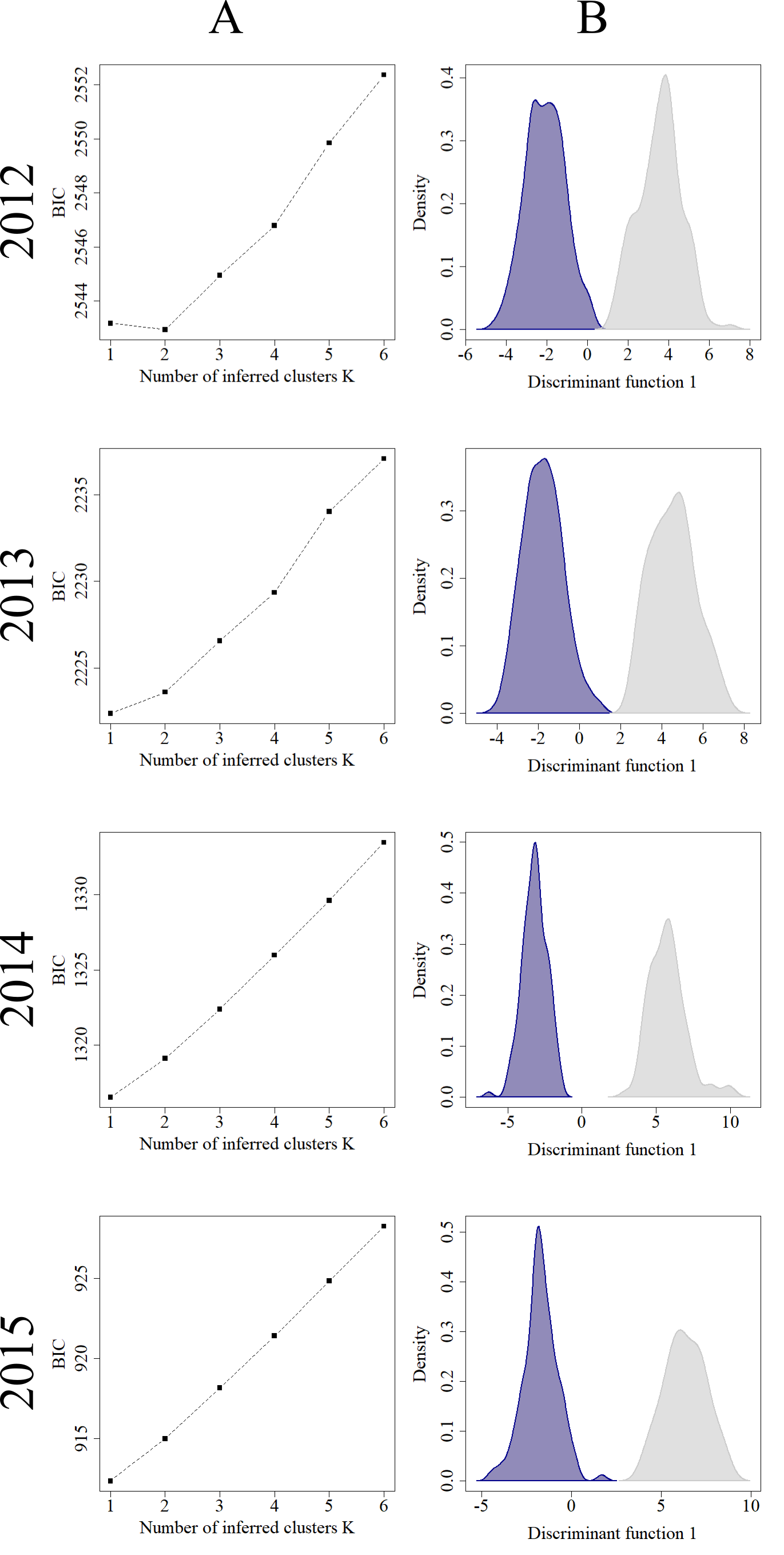


**Figure S3** Genetic clustering results from 2012 to 2015. A) Optimal number of genetic clusters in the spruce budworm population selected by Bayesian Information Criterion (*BIC*) for the DAPC and B) discriminant function for the *K* = 2 solution in 2012, 2013, 2014 and 2015. A *BIC* minimum was observed for *K* = 2 for 2012 suggesting that sampled larvae may be divided into two genetic clusters. The signal decreased and, from 2013 to 2015, increasing the number of clusters did not improve the *BIC* and the two clusters initially identified were no longer present.


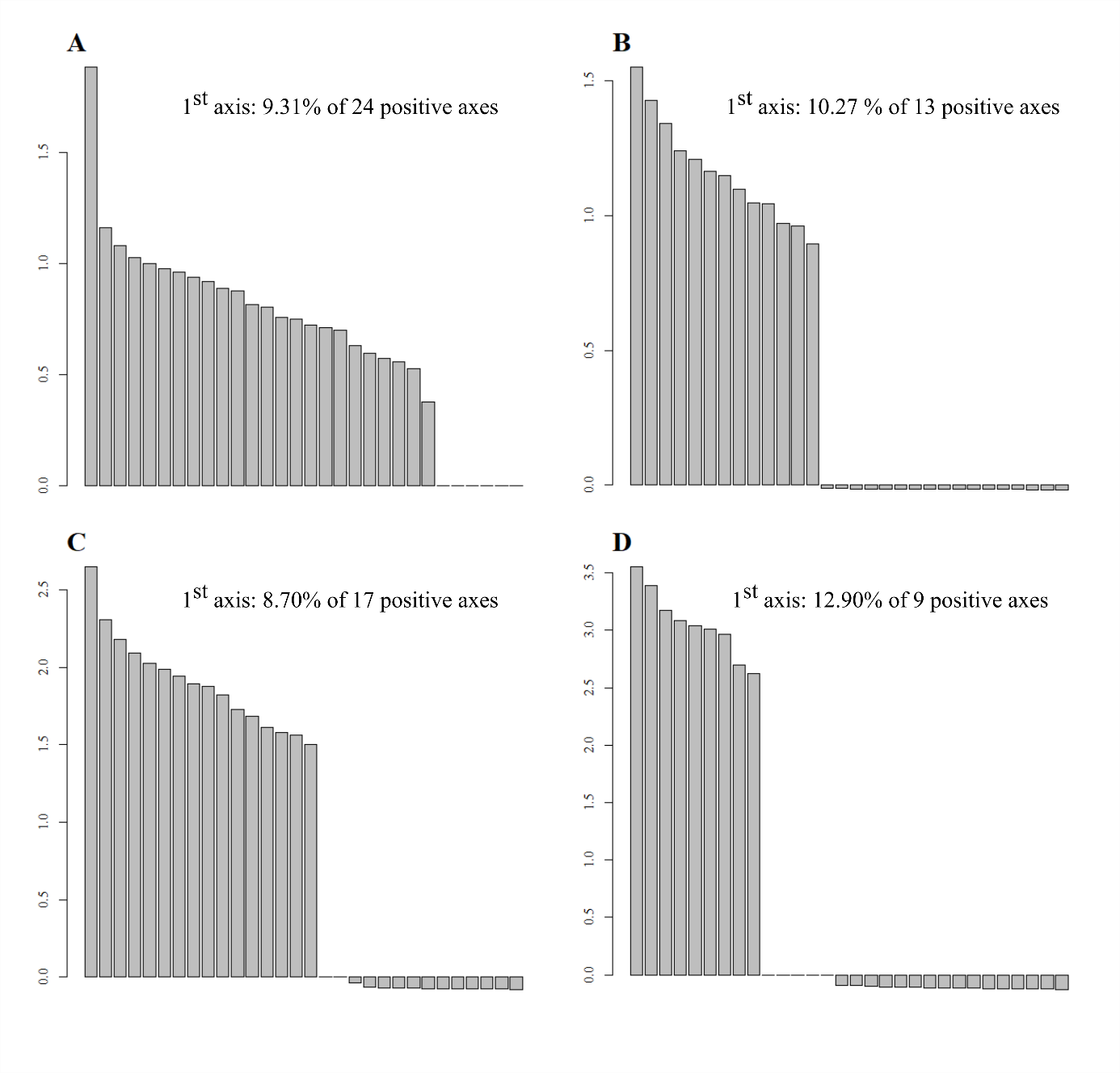


**Figure S4** Truncated barplot of the 30 first positive eigenvalues of the sPCA in A) 2012, B) 2013, C) 2014 and D) 2015. The first positive eigenvalue, which represents global spatial genetic structure, decreased in magnitude from 2012-2015 compared to the other eigenvalues. In 2012, the first positive axis represented 9.31% of the variance explained by the 24 positive axes, this percentage dropped to 12.90% of the variance explained by only 9 axes in 2015. This indicated a genetic homogenisation through the years.


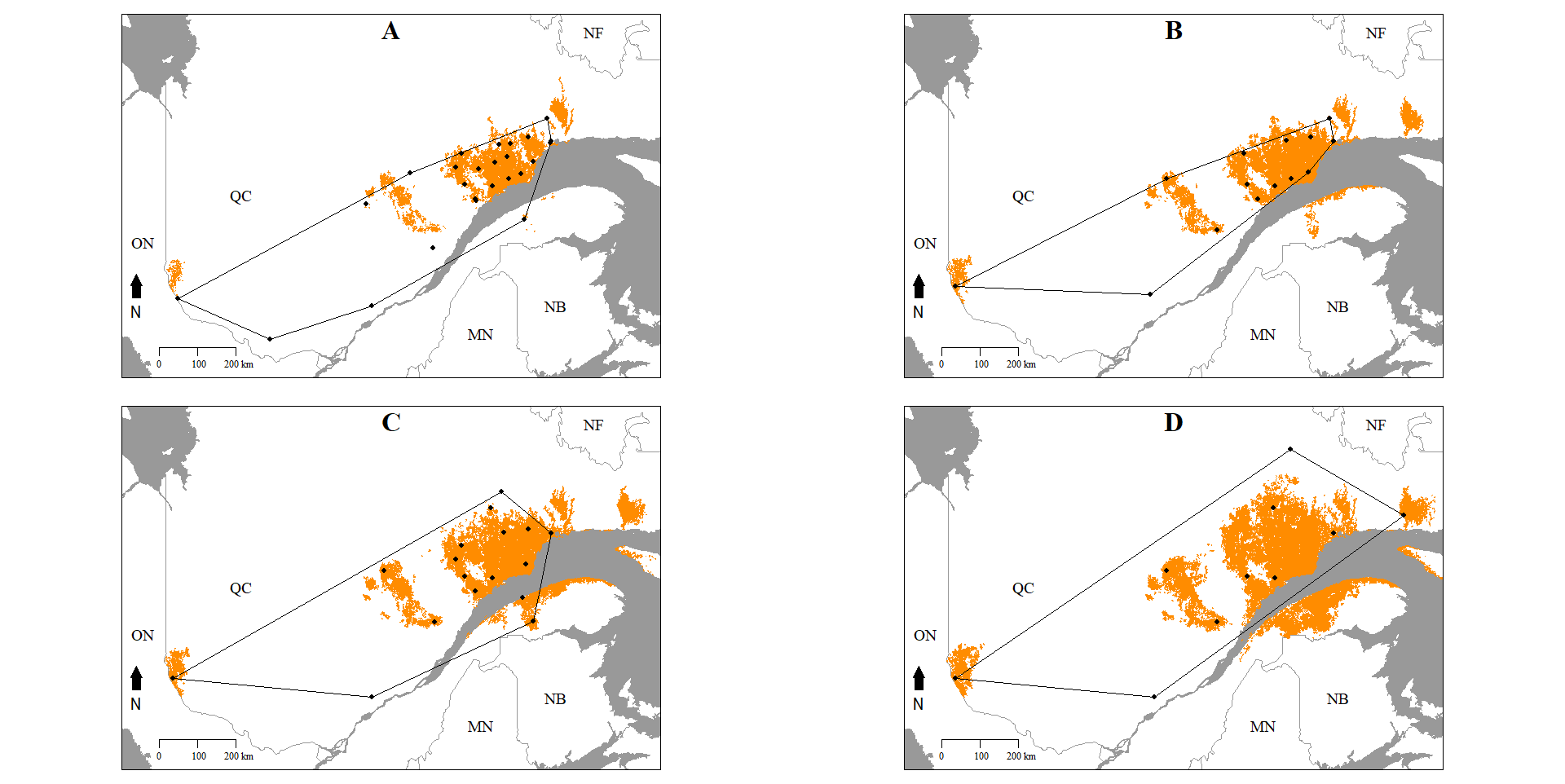


**Figure S5** Temporal evolution of the SBW defoliation areas in the Quebec from 2012 to 2015. Sampling sites (black dots), convex polygons encompassing all the sites sampled and spruce budworm defoliation areas (aerial inventories from the SOPFIM, www.sopfim.qc.ca,) were projected on the map of the study area for A) 2012, B) 2013, C) 2014, and D) 2015.


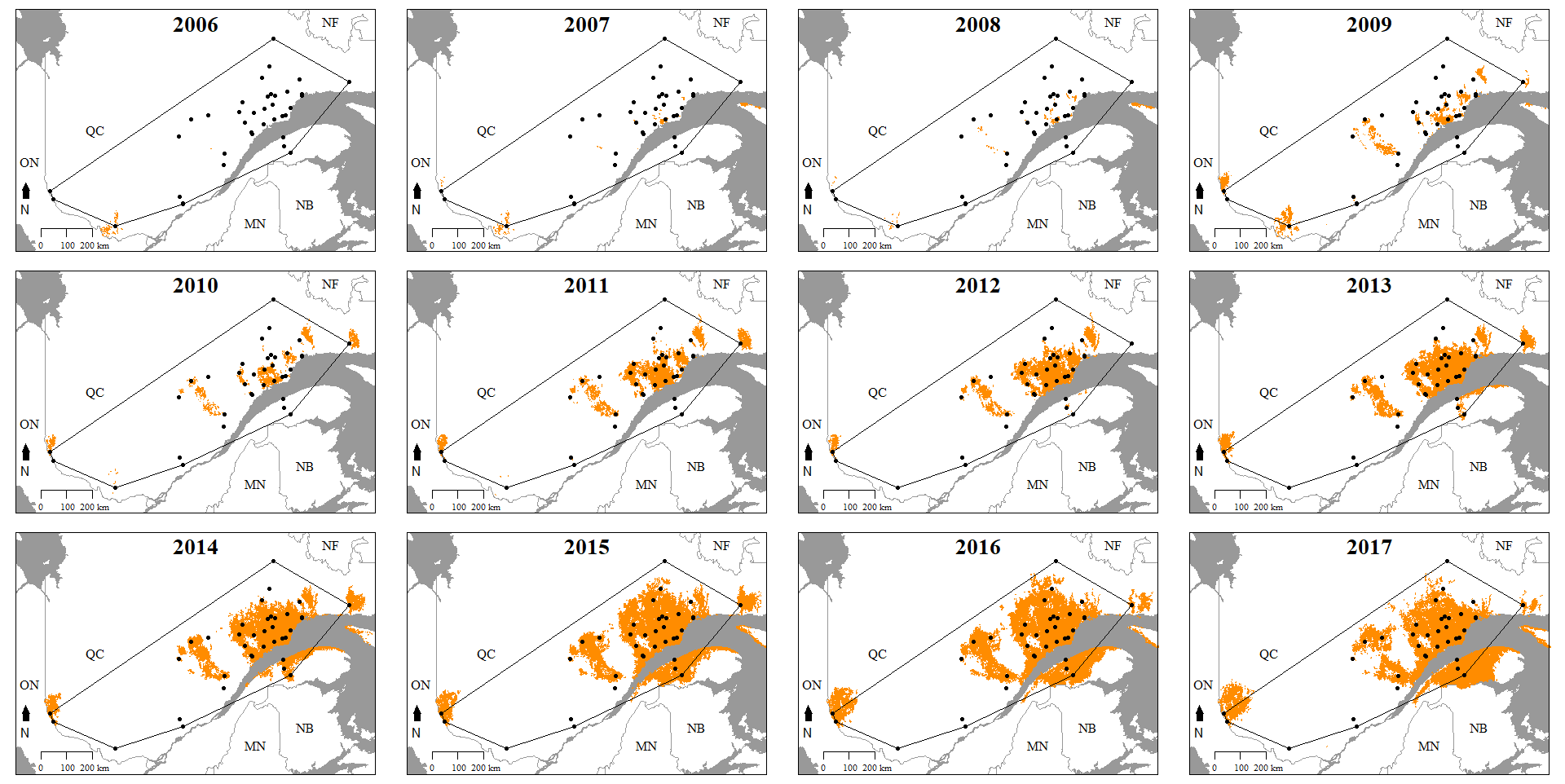


**Figure S6** Temporal evolution of the SBW defoliation areas in the Quebec from 2006 to 2017 Sites (black dots) sampled from 2012 to 2015, convex polygons encompassing all the sampling sites and spruce budworm defoliation map (aerial inventories from the SOPFIM, www.sopfim.qc.ca) in the Quebec region from 2006 to 2017, were projected on the map of the study area.


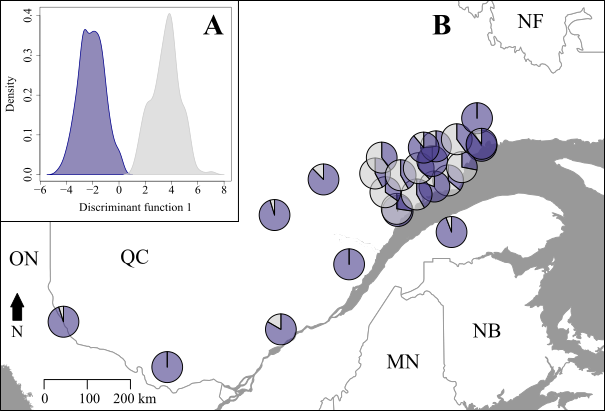


**Figure S7** Original figure of the DAPC 2012 results. A) Assignment of 2012 individuals by the Discriminant Analysis of Principal Components (DAPC) in the two genetic clusters in blue and grey and B) map of sampling sites displaying pie charts of the two groups membership. Each site is represented by a pie chart showing the proportion of individuals assigned to each of the two identified genetic clusters.


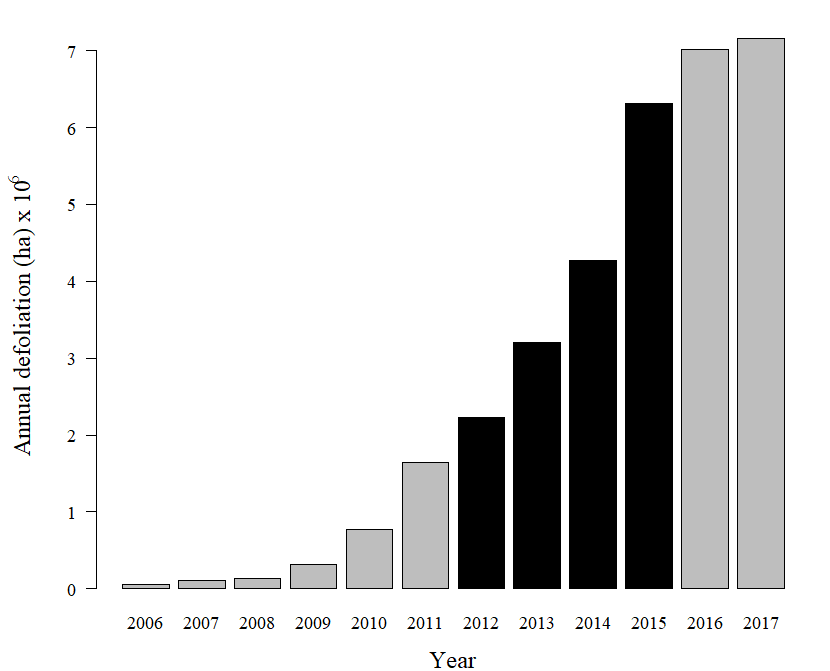


Figure S8 Annual SBW defoliation (in hectare) in Quebec from 2006 to 2017. The areas defoliated during the sampling period of this study are displayed in black.

**Table S1.** Summary of sample sites. In 2012, sites were selected to cover all the outbreak patches. The following years, some sites were discarded but close replacement sites were selected when possible. When substitutes sites were selected we considered them to be a single site through time (merged sites). Sample size (*n*), observed heterozygosity (*Ho*) (± SD), expected heterozygosity (*He*) (± SD), allelic richness (*Ar*) (± SD), total number of alleles (*n.all*), and *Fis* (± SD) per year and per sampling site.

| year | site | merged site | *n* | *Ho* | *He* | *Ar* | *n.all* | *Fis* |
| --- | --- | --- | --- | --- | --- | --- | --- | --- |
| 2012 | site_1 | site_1 | 25 | 0.155 ± 0.144 | 0.195 ± 0.142 | 1.688 ± 0.306 | 6821 | 0.186 ± 0.338 |
|  | site_2 | site_2 | 24 | 0.162 ± 0.144 | 0.207 ± 0.139 | 1.726 ± 0.277 | 6927 | 0.197 ± 0.346 |
|  | site_3 | site_3 | 16 | 0.163 ± 0.153 | 0.206 ± 0.149 | 1.718 ± 0.320 | 6701 | 0.184 ± 0.369 |
|  | site_4 | site_4 | 21 | 0.154 ± 0.144 | 0.196 ± 0.144 | 1.693 ± 0.312 | 6762 | 0.184 ± 0.351 |
|  | site_5 | site_5 | 21 | 0.147 ± 0.147 | 0.182 ± 0.147 | 1.646 ± 0.342 | 6594 | 0.171 ± 0.338 |
|  | site_6 | site_6 | 8 | 0.157 ± 0.185 | 0.200 ± 0.179 | 1.660 ± 0.448 | 6007 | 0.169 ± 0.428 |
|  | site_7 | site_7 | 17 | 0.165 ± 0.153 | 0.204 ± 0.148 | 1.712 ± 0.320 | 6713 | 0.163 ± 0.354 |
|  | site_8 | site_8 | 12 | 0.155 ± 0.162 | 0.194 ± 0.158 | 1.675 ± 0.379 | 6365 | 0.168 ± 0.385 |
|  | site_9 | site_9 | 24 | 0.156 ± 0.144 | 0.197 ± 0.141 | 1.700 ± 0.297 | 6845 | 0.189 ± 0.340 |
|  | site_10 | site_10 | 22 | 0.192 ± 0.149 | 0.223 ± 0.135 | 1.775 ± 0.241 | 7017 | 0.131 ± 0.313 |
|  | site_11 | site_11 | 23 | 0.186 ± 0.146 | 0.222 ± 0.133 | 1.774 ± 0.234 | 7040 | 0.151 ± 0.324 |
|  | site_12 | site_12 | 19 | 0.188 ± 0.151 | 0.224 ± 0.139 | 1.774 ± 0.257 | 6959 | 0.149 ± 0.332 |
|  | site_13 | site_13 | 25 | 0.171 ± 0.144 | 0.216 ± 0.137 | 1.754 ± 0.249 | 7014 | 0.192 ± 0.342 |
|  | site_15 | site_14 | 25 | 0.177 ± 0.144 | 0.220 ± 0.135 | 1.764 ± 0.242 | 7024 | 0.183 ± 0.337 |
|  | site_17 | site_15 | 10 | 0.165 ± 0.167 | 0.210 ± 0.161 | 1.723 ± 0.375 | 6421 | 0.175 ± 0.398 |
|  | site_18 | site_16 | 24 | 0.171 ± 0.140 | 0.217 ± 0.133 | 1.761 ± 0.248 | 7011 | 0.195 ± 0.338 |
|  | site_19 | site_17 | 24 | 0.181 ± 0.142 | 0.220 ± 0.133 | 1.767 ± 0.242 | 7020 | 0.166 ± 0.324 |
|  | site_20 | site_18 | 24 | 0.191 ± 0.146 | 0.218 ± 0.135 | 1.762 ± 0.242 | 7035 | 0.114 ± 0.297 |
|  | site_21 | site_19 | 10 | 0.158 ± 0.168 | 0.201 ± 0.165 | 1.689 ± 0.395 | 6296 | 0.172 ± 0.396 |
|  | site_22 | site_20 | 15 | 0.157 ± 0.153 | 0.203 ± 0.151 | 1.707 ± 0.331 | 6649 | 0.191 ± 0.378 |
|  | site_25 | site_21 | 25 | 0.174 ± 0.141 | 0.214 ± 0.135 | 1.752 ± 0.250 | 7012 | 0.174 ± 0.326 |
|  | site_27 | site_22 | 25 | 0.178 ± 0.142 | 0.216 ± 0.135 | 1.756 ± 0.246 | 7020 | 0.158 ± 0.318 |
|  | site_28 | site_23 | 7 | 0.159 ± 0.185 | 0.200 ± 0.181 | 1.670 ± 0.470 | 5950 | 0.155 ± 0.420 |
|  | site_29 | site_24 | 9 | 0.167 ± 0.171 | 0.198 ± 0.164 | 1.692 ± 0.408 | 6235 | 0.121 ± 0.364 |
|  | site_30 | site_25 | 9 | 0.159 ± 0.172 | 0.199 ± 0.169 | 1.682 ± 0.414 | 6195 | 0.160 ± 0.399 |
| 2013 | site_10 | site_10 | 25 | 0.208 ± 0.152 | 0.225 ± 0.132 | 1.781 ± 0.225 | 7061 | 0.082 ± 0.281 |
|  | site_11 | site_11 | 24 | 0.168 ± 0.139 | 0.204 ± 0.134 | 1.732 ± 0.261 | 6982 | 0.160 ± 0.321 |
|  | site_13 | site_13 | 28 | 0.184 ± 0.142 | 0.223 ± 0.133 | 1.772 ± 0.232 | 7051 | 0.168 ± 0.318 |
|  | site_16 | site_14 | 31 | 0.184 ± 0.142 | 0.218 ± 0.131 | 1.763 ± 0.228 | 7077 | 0.154 ± 0.308 |
|  | site_17 | site_15 | 27 | 0.179 ± 0.143 | 0.217 ± 0.133 | 1.762 ± 0.236 | 7040 | 0.168 ± 0.323 |
|  | site_18 | site_16 | 31 | 0.168 ± 0.137 | 0.208 ± 0.132 | 1.741 ± 0.242 | 7055 | 0.183 ± 0.319 |
|  | site_23 | site_20 | 18 | 0.181 ± 0.147 | 0.214 ± 0.139 | 1.755 ± 0.272 | 6911 | 0.138 ± 0.327 |
|  | site_27 | site_22 | 29 | 0.216 ± 0.153 | 0.227 ± 0.129 | 1.789 ± 0.208 | 7092 | 0.064 ± 0.266 |
|  | site_28 | site_23 | 28 | 0.176 ± 0.139 | 0.219 ± 0.132 | 1.765 ± 0.234 | 7059 | 0.184 ± 0.326 |
|  | site_29 | site_24 | 33 | 0.173 ± 0.137 | 0.214 ± 0.130 | 1.755 ± 0.229 | 7087 | 0.185 ± 0.317 |
|  | site_31 | site_3 | 32 | 0.174 ± 0.140 | 0.218 ± 0.130 | 1.764 ± 0.227 | 7071 | 0.198 ± 0.330 |
|  | site_32 | site_2 | 16 | 0.214 ± 0.167 | 0.228 ± 0.141 | 1.785 ± 0.263 | 6922 | 0.067 ± 0.307 |
|  | site_35 | site_8 | 32 | 0.177 ± 0.140 | 0.210 ± 0.131 | 1.745 ± 0.236 | 7067 | 0.150 ± 0.298 |
|  | site_36 | site_6 | 32 | 0.177 ± 0.136 | 0.216 ± 0.129 | 1.762 ± 0.227 | 7068 | 0.173 ± 0.310 |

**Table S1. (continued)** Summary of sample sites. In 2012, sites were selected to cover all the outbreak patches. The following years, some sites were discarded but close replacement sites were selected when possible. When substitutes sites were selected we considered them to be a single site through time (merged sites). Sample size (*n*), observed heterozygosity (*Ho*) (± SD), expected heterozygosity (*He*) (± SD), allelic richness (*Ar*) (± SD), total number of alleles (*n.all*), and *Fis* (± SD) per year and per sampling site.

| year | site | merged site | *n* | *Ho* | *He* | *Ar* | *n.all* | *Fis* |
| --- | --- | --- | --- | --- | --- | --- | --- | --- |
| 2014 | site_10 | site_10 | 9 | 0.170 ± 0.171 | 0.218 ± 0.163 | 1.744 ± 0.381 | 6414 | 0.179 ± 0.407 |
|  | site_11 | site_11 | 16 | 0.160 ± 0.152 | 0.194 ± 0.148 | 1.690 ± 0.331 | 6644 | 0.150 ± 0.345 |
|  | site_12 | site_12 | 15 | 0.179 ± 0.152 | 0.224 ± 0.145 | 1.770 ± 0.287 | 6835 | 0.177 ± 0.361 |
|  | site_13 | site_13 | 12 | 0.168 ± 0.157 | 0.207 ± 0.151 | 1.731 ± 0.337 | 6602 | 0.156 ± 0.367 |
|  | site_16 | site_14 | 15 | 0.181 ± 0.153 | 0.226 ± 0.144 | 1.777 ± 0.282 | 6848 | 0.177 ± 0.367 |
|  | site_18 | site_16 | 17 | 0.168 ± 0.148 | 0.216 ± 0.142 | 1.755 ± 0.283 | 6859 | 0.197 ± 0.365 |
|  | site_23 | site_20 | 11 | 0.168 ± 0.161 | 0.209 ± 0.154 | 1.732 ± 0.353 | 6529 | 0.162 ± 0.379 |
|  | site_24 | site_26 | 14 | 0.165 ± 0.153 | 0.205 ± 0.149 | 1.724 ± 0.324 | 6674 | 0.170 ± 0.360 |
|  | site_26 | site_27 | 12 | 0.169 ± 0.161 | 0.209 ± 0.156 | 1.724 ± 0.345 | 6564 | 0.160 ± 0.377 |
|  | site_27 | site_22 | 12 | 0.160 ± 0.157 | 0.198 ± 0.155 | 1.698 ± 0.360 | 6480 | 0.163 ± 0.368 |
|  | site_29 | site_24 | 12 | 0.164 ± 0.159 | 0.204 ± 0.154 | 1.715 ± 0.351 | 6528 | 0.161 ± 0.373 |
|  | site_31 | site_3 | 10 | 0.174 ± 0.163 | 0.217 ± 0.158 | 1.747 ± 0.359 | 6508 | 0.157 ± 0.378 |
|  | site_33 | site_2 | 14 | 0.165 ± 0.150 | 0.209 ± 0.147 | 1.737 ± 0.311 | 6731 | 0.175 ± 0.366 |
|  | site_35 | site_8 | 14 | 0.163 ± 0.151 | 0.205 ± 0.149 | 1.721 ± 0.326 | 6661 | 0.173 ± 0.366 |
|  | site_36 | site_6 | 15 | 0.176 ± 0.151 | 0.223 ± 0.144 | 1.770 ± 0.282 | 6849 | 0.185 ± 0.366 |
|  | site_37 | site_28 | 15 | 0.179 ± 0.153 | 0.222 ± 0.145 | 1.767 ± 0.286 | 6839 | 0.169 ± 0.359 |
|  | site_38 | site_29 | 10 | 0.160 ± 0.160 | 0.204 ± 0.160 | 1.715 ± 0.376 | 6407 | 0.172 ± 0.389 |
| 2015 | site_11 | site_11 | 19 | 0.170 ± 0.143 | 0.218 ± 0.139 | 1.760 ± 0.267 | 6922 | 0.198 ± 0.350 |
|  | site_14 | site_30 | 14 | 0.164 ± 0.152 | 0.209 ± 0.146 | 1.736 ± 0.316 | 6706 | 0.186 ± 0.379 |
|  | site_18 | site_16 | 19 | 0.160 ± 0.145 | 0.207 ± 0.140 | 1.733 ± 0.283 | 6881 | 0.200 ± 0.363 |
|  | site_24 | site_26 | 19 | 0.175 ± 0.145 | 0.223 ± 0.139 | 1.771 ± 0.260 | 6954 | 0.188 ± 0.346 |
|  | site_29 | site_24 | 15 | 0.175 ± 0.155 | 0.224 ± 0.147 | 1.765 ± 0.292 | 6817 | 0.189 ± 0.372 |
|  | site_31 | site_3 | 15 | 0.176 ± 0.152 | 0.222 ± 0.147 | 1.762 ± 0.293 | 6817 | 0.180 ± 0.364 |
|  | site_33 | site_2 | 15 | 0.177 ± 0.154 | 0.223 ± 0.146 | 1.764 ± 0.297 | 6795 | 0.182 ± 0.369 |
|  | site_34 | site_31 | 13 | 0.168 ± 0.155 | 0.206 ± 0.148 | 1.732 ± 0.325 | 6657 | 0.160 ± 0.367 |
|  | site_35 | site_8 | 14 | 0.176 ± 0.155 | 0.219 ± 0.148 | 1.758 ± 0.304 | 6761 | 0.167 ± 0.369 |
|  | site_36 | site_6 | 12 | 0.174 ± 0.159 | 0.211 ± 0.150 | 1.742 ± 0.331 | 6634 | 0.149 ± 0.367 |
